# Small forces that differ with prior motor experience can communicate movement goals during human-human physical interaction

**DOI:** 10.1186/s12984-017-0217-2

**Published:** 2017-01-31

**Authors:** Andrew Sawers, Tapomayukh Bhattacharjee, J. Lucas McKay, Madeleine E. Hackney, Charles C. Kemp, Lena H. Ting

**Affiliations:** 1 Department of Kinesiology and Nutrition, University of Illinois at Chicago, Chicago, IL 60612 USA; 2 W.H. Coulter Department of Biomedical Engineering, Emory University and Georgia Institute of Technology, 1760 Haygood Drive Suite W 200, Atlanta, GA 30322-4250 USA; 3 Atlanta VA Center for Visual and Neurocognitive Rehabilitation, Atlanta, GA 30033 USA; 4 Department of Medicine, Division of General Medicine and Geriatrics, Emory University School of Medicine, Atlanta, GA 30322 USA; 5 Department of Rehabilitation Medicine, Division of Physical Therapy, Emory University School of Medicine, Atlanta, GA 30322 USA

**Keywords:** Rehabilitation, Human-human interaction, Haptics, Rehabilitation robotics, Human-robot interaction

## Abstract

**Background:**

Physical interactions between two people are ubiquitous in our daily lives, and an integral part of many forms of rehabilitation. However, few studies have investigated forces arising from physical interactions between humans during a cooperative motor task, particularly during overground movements. As such, the direction and magnitude of interaction forces between two human partners, how those forces are used to communicate movement goals, and whether they change with motor experience remains unknown. A better understanding of how cooperative physical interactions are achieved in healthy individuals of different skill levels is a first step toward understanding principles of physical interactions that could be applied to robotic devices for motor assistance and rehabilitation.

**Methods:**

Interaction forces between expert and novice partner dancers were recorded while performing a forward-backward partnered stepping task with assigned “leader” and “follower” roles. Their position was recorded using motion capture. The magnitude and direction of the interaction forces were analyzed and compared across groups (i.e. expert-expert, expert-novice, and novice-novice) and across movement phases (i.e. forward, backward, change of direction).

**Results:**

All dyads were able to perform the partnered stepping task with some level of proficiency. Relatively small interaction forces (10–30N) were observed across all dyads, but were significantly larger among expert-expert dyads. Interaction forces were also found to be significantly different across movement phases. However, interaction force magnitude did not change as whole-body synchronization between partners improved across trials.

**Conclusions:**

Relatively small interaction forces may communicate movement goals (i.e. “what to do and when to do it”) between human partners during cooperative physical interactions. Moreover, these small interactions forces vary with prior motor experience, and may act primarily as guiding cues that convey information about movement goals rather than providing physical assistance. This suggests that robots may be able to provide meaningful physical interactions for rehabilitation using relatively small force levels.

## Background

Physical human-human interactions (HHI) occur between two people working towards a common motor goal such as moving a table together or dancing with a friend. Physical HHI can also occur between two people with different motor abilities such as a physical therapist helping a patient learn to balance during rehabilitation. Despite the prevalence of HHI in our lives, interaction forces during cooperative motor tasks have only been characterized in a few studies [1–4], and never during overground walking. Moreover, how such forces differ between individuals of varying skill levels, such as a physical therapist and patient remain unknown [5]. As a first step we sought to quantify interaction force magnitude during HHI, and how they vary as function of movement goals and skill level during overground movement.

Principles of HHI could be used to guide the design and control of assistive and rehabilitative robots that physically interact with patients [6–10]. By studying how two unimpaired adult partners interact when performing a joint motor task, our goal is to reveal sensorimotor principles underlying intuitive physical interactions. Such principles of physical interaction could be used to improve the effectiveness of robots that provide permanent motor assistance or short-term rehabilitation for locomotor deficits [11, 12], while also reducing the training and adaptation required of the user [13].

While there have been a few previous studies of HHI, their results may not be applicable to physical HHI during overground walking [6, 14]. Although HHI research is often motivated by whole-body motor behaviors such as lifting a heavy object or dancing with a partner [1, 15], HHI is typically studied using seated visuomotor tasks, with one exception [16]. However, studies using single joint motions of the upper extremity [1, 9, 17–19] may not generalize to multi-joint whole-body movements while walking [20, 21]. Perhaps more importantly, prior studies focus on tasks requiring visual feedback [2, 22, 23], which may distort or minimize the potentially powerful role of haptic information during HHI tasks [14]. Further, prior studies do not define specific roles for each partner but allow them to spontaneously emerge [9, 18, 19, 23–28], which differs from many real life HHI scenarios relevant to motor assistance and rehabilitation (e.g., dance partners, therapist-patient, coach-athlete), where defined roles are established a priori. Moreover, there has been limited examination of how differences in prior motor experience or skill level affect the resulting interactions [3, 18, 29, 30]. How haptic information is used to coordinate HHI tasks may be better revealed by assigning fixed roles of “leader” (i.e. therapist, coach) and “follower” (i.e. patient, athlete) a priori, and by examining how differences in motor experience among the members of a dyad affect performance.

Here, we studied force interactions in HHI during a cooperative whole-body motor task based on partner dance. Physical interactions were limited to forces at the hands, and participants were required to perform the cooperative motor task in the absence of visual or auditory cues. Thus all communication between partners was required to occur through forceful interactions alone. We recruited experts, i.e. professional partner dancers, and novices to participate in the experiments as expert-expert, expert-novice, or novice-novice dyads. We measured whole-body position and interaction forces at the hands between partners as they performed a predictable and unpredictable partnered stepping task (PST). Our goal was to characterize the magnitude of the interaction forces during partnered stepping, and test whether interaction forces 1) differ with movement goals; 2) are altered by prior motor experience, or 3) change with short-term practice.

## Methods

### Participant recruitment

We recruited two cohorts of participants: trained expert and untrained novice partner dancers. Inclusion criteria for all participants included age greater than 18 years. Inclusion criteria for the trained experts included a minimum of 10 years of experience in partner dance [31], plus 2 years experience teaching partner dance. For all participants, exclusion criteria were medical conditions, assessed by self-report, that could result in impaired balance or sensory loss, including significant musculoskeletal, neurologic, or cardiopulmonary conditions. For untrained novices, exclusion criteria included any formal training in partner dance. Each expert and novice participant was randomized into one of three groups: Expert leader-Expert follower (EE), Expert leader-Novice follower (EN), or Novice leader-Novice follower (NN). Written, informed consent was obtained from each subject. The Institutional Review Board of the Georgia Institute of Technology approved all protocols.

### Experimental protocol

To characterize the interaction forces between human partners during a cooperative whole-body motor task we studied a partnered stepping task (PST) based on principles of partner dance. Partnered stepping offers several key features that make it an ideal experimental model to study whole-body cooperative human-human interaction. First, interaction forces alone are sufficient for communication during partner dance [15]. This allows the task to be performed in the absence of vision, avoiding the potential confound of participants using visual information to coordinate and interpret movements. Secondly, there are explicit movement goals that can be used to evaluate motor performance. For example, metrics of whole-body synchronization can be used to quantify differences in performance between groups. Lastly, by recruiting “expert” and “novice” performers, differences in physical interaction owing to motor experience can be readily examined.

### The Partnered Stepping Task (PST)

All dyads performed a predictable and an unpredictable PST. The role of each participant as a leader or follower was determined prior to each experiment. In expert-novice dyads, the expert was always the leader; in matched dyads i.e. expert-expert and novice-novice, the leader was chosen randomly. A week before the experiment, the leader of each dyad received instructional videos of the PST. This allowed the leader to practice and become familiar with each of the three stepping patterns used in the PST prior to the experiment. Each leader was required to demonstrate proficiency in correctly performing the unpredictable PST before the start of the experiment. In contrast, followers were not given any instruction as to the steps involved in the PST. They were only told to follow the movements of the leader based on their sensing and perception of the interaction forces transmitted through instrumented force handles held by each partner. Specifically, they were told to maintain their position relative to the leader based on the direction and magnitude of the perceived force.

All participants wore blindfolds and earphones to restrict visual and auditory feedback that could otherwise aid them in synchronizing movements with their partners, and confound the role and analyses of the interaction forces. To help maintain a consistent tempo (i.e. step frequency) across trials and between dyads, leaders in each dyad received auditory cues at 126 beats per minutes (bpm) via headphones. The leaders were instructed to step on every second beat (i.e. 63 bpm). The followers did not receive any auditory cues but were required to listen to white noise through their earphones.

### Predictable PST

**Fig. 1:**
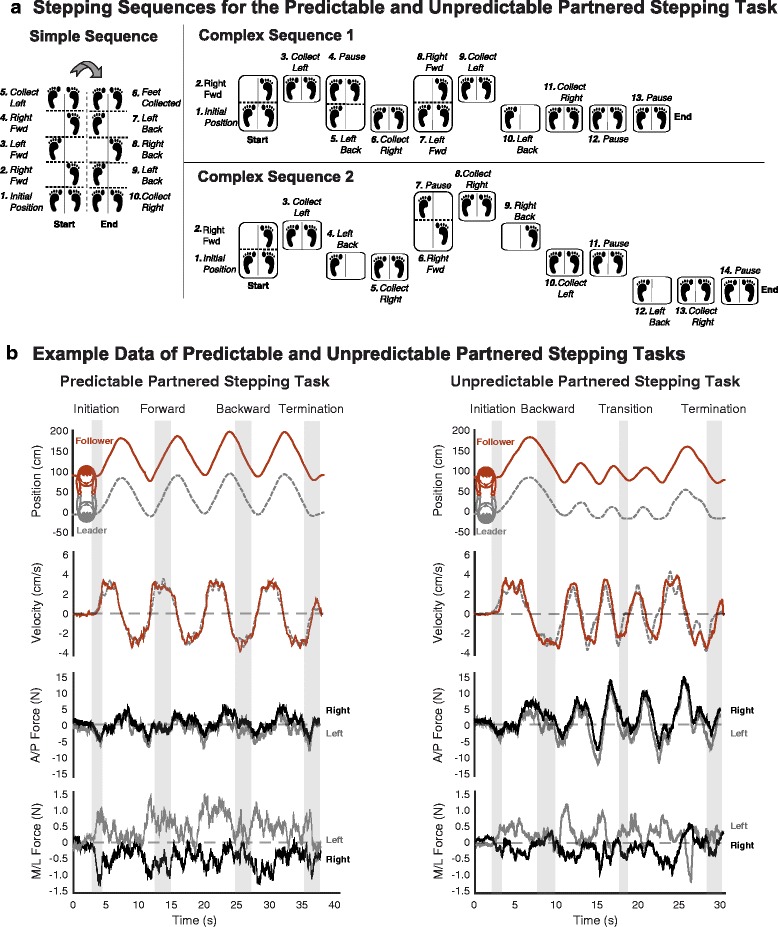
An illustration of the stepping sequences from the perspective of the leader, and example movement and force data during the partnered stepping task (PST). **a** The simple stepping task consisted for three steps forward, collect feet together, three steps backward, collect feet together. The simple sequence was repeated four times during the predictable PST trials. Complex sequence 1 and 2 were created using a pseudorandom sequence generator to decrease the likelihood that the follower could anticipate the step. The unpredictable PST trials consisted of performing the two complex sequences and the simple sequence in a random order as prescribed by the experimenter to the leader before each unpredictable trial. To control step frequency the leaders in each dyad received auditory cues of a consistent beat at 126 beats per minutes (bpm) via their headphones. The leaders were instructed to step on every second beat (i.e. 63 bpm). The followers did not receive any auditory cues. **b** Example position and velocity data of a leader (grey, dashed line) and follower (red, solid line), as well as interaction forces between their right (black line) and left (grey line) hands during a predictable and unpredictable stepping sequence. Velocity data of the follower was used to identify movement phases (grey boxes). The axial (A/P) forces were similar between the left and right hands. This was consistent across all dyads regardless of composition (i.e. expert or novice). Therefore, only the axial interaction force from the right hand of the leader/left hand of the follower was analyzed. The medial-lateral (M/L) forces were of small magnitude during the predictable and unpredictable PST. As a result they were not analyzed in detail

The predictable PST involved performing four repetitions of a simple stepping sequence that consisted of three steps forward and three steps backward (Fig. 1a). The predictable PST was selected to emulate a simple forward/backward partner dance step. All dyads performed 4 trials of the predictable stepping pattern.

### Unpredictable PST

The unpredictable PST involved performing two complex stepping patterns and one simple stepping pattern in a random order. The unpredictable PST was specifically designed to be unfamiliar to partner dancers, so as to necessitate greater reliance on interaction forces for successful cooperation between participants. The unpredictable PST was selected to approximate “dance-like” interactions between two human partners in a controlled and reproducible manner. Because the sequences of the unpredictable PST were not a standard pattern of choreography from any form of partnered dance, we did not expect experts to be overtly familiar with it. The two complex stepping patterns were created using a pseudorandom sequence generator where ‘0’ represents ‘no movement’, ‘1’ represents ‘move forward’, and ‘2’ represents ‘move backward’ (Fig. 1a). Prior to each trial, the order in which the two complex stepping patterns and simple stepping pattern were to be performed was communicated the leader but not the follower via a microphone that was connected to the earphones worn by the leader. All dyads performed 10 trials of the unpredictable PST.

### Data collection and processing

**Fig. 2:**
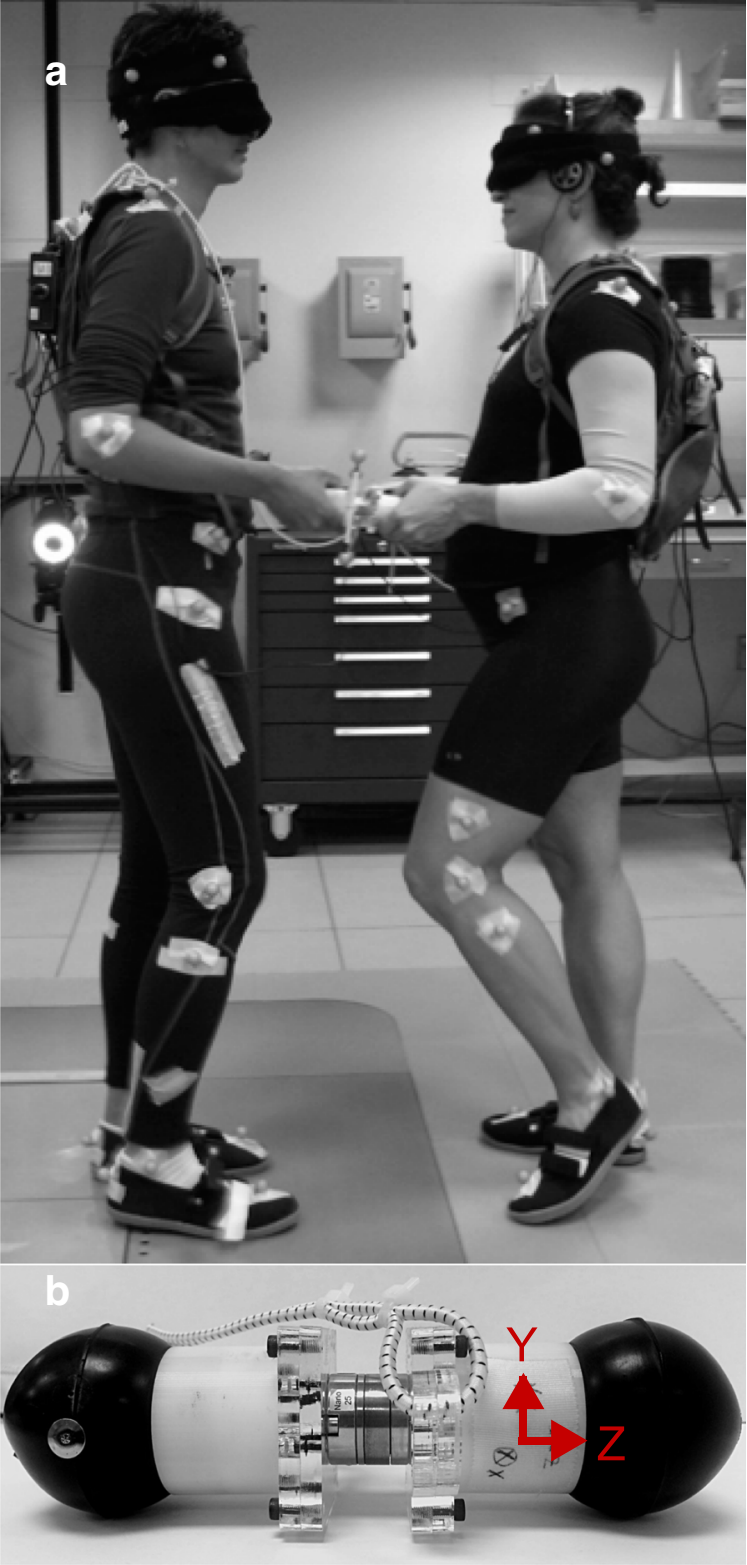
Experimental setup. **a**: Image of an example dyad for the partnered stepping task; leader on the right, and follower on the left. **b**: Custom-built force-sensing device with a 6-axis load cell in the center, spherical rubber handle attached on each end, and the directions of the recorded forces

To characterize the interaction forces between leaders and followers during the PST left and right interaction forces were collected at 1080 Hz using custom-built force-sensing devices, one in each hand. Each device (Fig. 2) consisted an ATI Nano-25 six axis force/torque (F/T) transducer with a spherical rubber handle attached to each side. The force sensors had a specified linear force sensing range of at least ± 125 N (ATI Industrial Automation, Apex, NC). Each force transducer was calibrated with known loads prior to data collection, and baseline voltage levels were recorded that were subtracted from the collected data. Force data was low-pass filtered using a 3rd order 60Hz Butterworth filter and then transformed from local to laboratory coordinate system using custom MATLAB™ (MathWorks, Natick, MA) code.

To monitor the position of the leader and the follower during the PST, three-dimensional marker coordinate data from a custom whole-body 20-marker set were collected at 120 Hz using an eight-camera Vicon motion capture system (Centennial, CO). Gaps in marker coordinate data due to occlusions were interpolated with a linear interpolation function, and then filtered with a 3^rd^ order 20 Hz low-pass Butterworth filter. All post-processing was done using custom MATLAB™ (MathWorks, Natick, MA) code.

### Data analyses

Whole-body position of the leader and follower was approximated based on the positions of the marker located on the 7^th^ cervical (C7) vertebrae. The leader’s trajectory was selected rather than the follower’s because it more closely represented the intended direction of movement of each dyad. The C7 vertebrae provided the most reliable estimate of body movement direction and had fewer occlusions than lower-body markers. Whole-body rather than foot motion was selected to identify movement phases because of its consistency and ability to differentiate movement phases in the same direction (i.e. initiation versus forward). Specific movement phases of the PST (i.e. initiation, forward, backward, and change of direction) were identified based on the estimated velocity of the leader, obtained by differentiating the estimated position trajectory using a smoothing Savitzky-Golay differentiating filter. The initiation of the PST was identified as the first peak in the velocity trajectory of the leader. Forward and backward movements were identified as the periods of near constant positive and negative velocity, respectively. Changes in direction were identified as the period of time between the velocity peaks immediately before and after zero crossings of the leader’s velocity trajectory (Fig. 1b).

To broadly characterize the magnitude of the interaction forces used during the PST, we calculated the range of interaction forces as well as the mean of the peak interaction force across trials during the predictable and unpredictable PST. To test whether the peak interaction force was affected by motor experience differences between the EE, EN, and NN dyads were tested with a 1-way ANOVA (α = 0.05). Multiple comparisons were adjusted for with Tukey post-hoc tests.

To characterize interaction forces during specific movement phases among EE, EN, and NN dyads we calculated the mean force used during each movement phase (i.e. initiation, forward, backward, change of direction) of the predictable and unpredictable PST. This was done by indexing the recorded interaction force data according to the time points of the movement phases identified above in a semi-automated fashion that was verified visually. To test whether interaction forces contain information about movement goals, we compared interaction force magnitude across movement phases (i.e. initiation, forward, backward, and change of direction) within each group (i.e. *within group repeated measures factor, 4-levels*). To test whether motor experience (i.e. *EE* versus *EN* versus *NN*) modifies the interaction forces used across movement goals the interaction force for each movement phase was compared between groups (i.e. *between groups factor 3-levels*). These within group and between group comparisons were tested with a 2-way mixed-design ANOVA (α = 0.05). Multiple comparisons were adjusted for with a Bonferroni correction. All statistical analyses were performed using SPSS (Chicago, IL).

To characterize the performance of the unpredictable PST whole-body synchronization between the leader and follower was quantified for each trial as the spatial error between partners. Spatial error was calculated as the mean value of the absolute difference in the anterior-posterior position between the leader and follower with respect to the initial distance between them. To test whether performance of the unpredictable PST improved with practice the mean spatial error was compared between the first and last trials for each dyad using two-sided paired t-tests (α = 0.05). To test whether interaction forces changed with practice we compared the range of interaction forces between the first and last trial within each group using two-sided paired t-tests (α = 0.05).

## Results

### Participant characteristics

Eighteen expert dancers (nine females, 34 ± 0.92 years old; nine males 36 ± 0.84 years old) (mean ± 1SE) and 24 novice dancers (nine female, 21 ± 0.26 years old; 18 males 23 ± 0.16 years old) were recruited and allocated into Expert-Expert (EE, *n* = 6), Expert-Novices (EN, *n* = 8), and Novice-Novice (NN, *n* = 8) dyads in the experiment. All expert partner dancers had a minimum of 10 years of partner dance experience (average of 15 ± 7.9 years) as well as at least 2 years of partner dance instruction experience. Average height (experts: 1.72 ± 0.02 m; novices: 1.73 ± 0.02 m) and weight (experts: 72 ± 3.1 kg; novices: 70 ± 2.4 kg), did not differ between groups (*p* > 0.05).

### Reporting of interaction forces

Only forces in the direction of motion (i.e. anterior-posterior, Z-axis in Fig. 2b) were analyzed in detail. Medial-lateral forces were found to be 10–20 times smaller in magnitude than anterior-posterior forces, and changed little during the performance of the partnered stepping task (Fig. 1b). Additionally, while interaction forces were recorded in the left and right hands, we have focused our analyses on the forces from the right hand of the leader/left hand of the follower.

### Range and mean peak interaction force

**Fig. 3:**
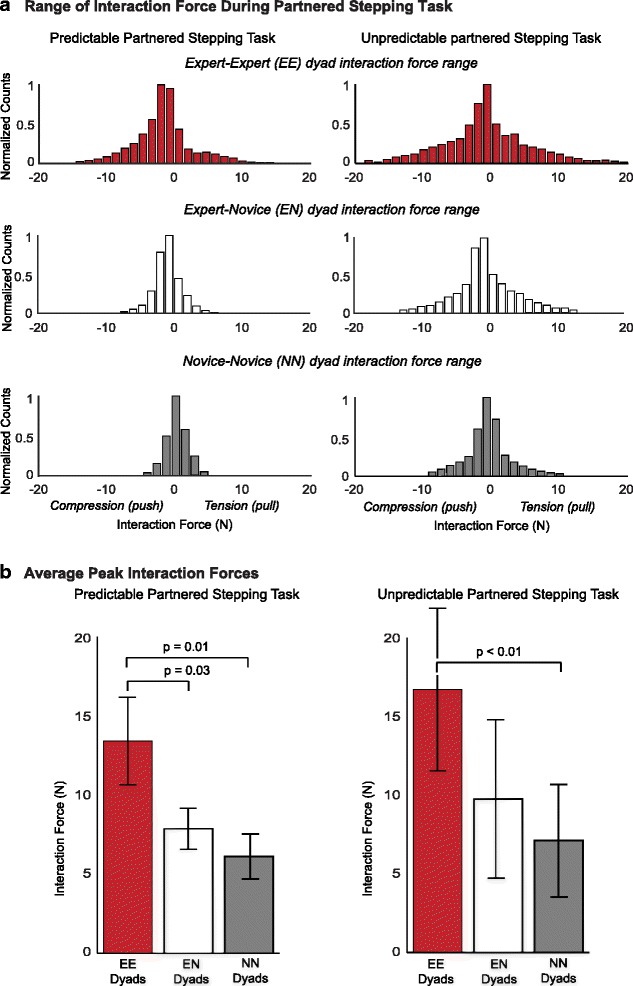
Interaction forces during the predictable and unpredictable partnered stepping task (PST). **a** Histograms depicting the distribution of the interaction forces observed during the predictable (*left*) and unpredictable (*right*) PST. The distribution of observed interaction forces was larger during the unpredictable than predictable PST, as well as for dyads with an expert leader regardless of the version of the PST. **b** The difference in the distribution of the histograms is reflected in the differences of the mean of the peak interaction force across trials between groups. While larger peak interaction forces were observed in EE dyads than the EN and NN dyads during the predictable and unpredictable PST, only the difference between the EE and NN dyads was found to be significant (*p* = 0.01)

Among the small range of interaction forces observed during the predictable and unpredictable PST, those used by experts were larger (Fig. 3a). Additionally, forces during the unpredictable PST were generally larger than those in the predictable PST. During the predictable PST there was a statistically significant difference between dyads in peak interaction forces as determined by one-way ANOVA (*F* (2, 20) = 8.41, *p* < 0.01). Post-hoc testing revealed that EE dyads had significantly larger peak interaction forces (mean ± SE) (12.3 ± 2.76 N) than the NN dyads (5.86 ± 0.96 N), *p* < 0.01 and the EN dyads (7.21 ± 1.27 N), *p* = 0.03. There was no significant difference in interaction force magnitude between the EN and NN dyads (*p* = 0.51). During the unpredictable PST significant differences were observed in peak interaction force magnitude across dyads (*F* (2, 20) = 3.55, *p* = 0.04). Post-hoc testing revealed that EE dyads had significantly larger peak interaction forces (mean ± SE) (15.84 ± 5.43 N) than the NN dyads (6.53 ± 3.28 N), *p* = 0.01 but not EN (9.95 ± 5.89 N), *p* = 0.11 (Fig. 3b). As with the predictable PST interaction peak interaction force magnitude was not significantly different between the EN and NN dyads (*p* = 0.46).

### Within and between group differences in interaction forces

Interaction forces differed within and between movement phases as a function of prior motor experience. A Two-way mixed ANOVA revealed a statistically significant interaction between group (Expert-Expert: EE, Expert-Novice: EN, and Novice-Novice: NN) and movement phase on interaction force magnitude during the predictable (*F* (6,57) = 7.92, *p* < 0.001) and unpredictable (*F* (6,57) = 52.22, *p* < 0.001) PST. Given this interaction, simple effects rather than main effects will be reported below for the predictable and unpredictable PST. Mauchly’s test of sphericity indicated that during the predictable and unpredictable PST the assumption of sphericity was met for the interaction between group and movement phase, predictable: *X* ^2^ (5) = 10.84, *p* = 0.07, unpredictable: *X* ^2^ (5) = 1.80, *p* = 0.876.

Expert-Expert dyads used significantly larger interaction forces within movement phases of the predictable PST than Expert-Novice or Novice-Novice dyads (Table 1). With the exception of the backward movement phase (*F* (2,19) = 3.32, *p* = 0.06), there were statistically significant differences in force magnitude between groups during the initiation (*F* (2,19) = 8.01, *p* = 0.003), forward (*F* (2,19) = 7.36, *p* = 0.004), and changes of direction (*F* (2,19) = 8.73, *p* = 0.002) movement phases. Post-hoc testing revealed that during initiation interaction forces (mean ± SD) were significant smaller in the EN (−2.59 ± 1.29 N, *p* = 0.017) and NN (−1.81 ± 0.99 N, *p* = 0.003) dyads compared to EE dyads (−5.27 ± 2.09 N). There was no significant difference between EN and NN dyads (*p* = 0.660). During forward stepping interaction forces were significantly smaller in the EN (−1.43 ± 0.90 N, *p* = 0.017), and NN dyads (0.84 ± 1.09 N, *p* = 0.005) compared to EE dyads (−4.17 ± 1.09 N). There was no significant difference between EN and NN dyads (*p* = 0.802). While changing direction interaction forces were significantly smaller in the EN (−0.66 ± 0.99 N, *p* = 0.021) and NN (0.43 ± 0.69 N, *p* = 0.002) dyads compared to the EE dyads (−3.18 ± 0.99 N). There was no significant difference between EN and NN dyads (*p* = 0.437).

**Table 1:** Mean interaction force per movement phase during the predictable partnered stepping task

	Movement phases			
	Initiation (INT)	Forward (FWD)	Backward (BKW)	Change Direction (CD)
Expert-Expert (EE)	-5.27 ± 2.09 N	-4.17 ± 1.09 N	3.38 ± 1.29 N	-3.18 ± 0.99 N
Expert-Novice (EN)	-2.59 ± 1.29 N	-1.43 ± 0.90 N	2.49 ± 1.69 N	-0.66 ± 0.99 N
Novice-Novice (NN)	-1.81 ± 0.99 N	-0.84 ± 1.09 N	1.99 ± 1.69 N	0.43 ± 0.69 N

Negative values are compression and positive values are tension

See text for significance values

Interaction forces differed between some but not all movement phases among all groups during the predictable PST. There were statistically significant effects of movement phase on interaction force magnitude in the EE (*F* (3,15) = 31.03, *p* < 0.0005), EN (*F* (3,21) = 30.96, *p* < 0.0005) and NN dyads (*F* (3,21) = 47.56, *p* < 0.0005). Pairwise comparisons with Bonferroni corrections revealed that among all groups interaction force magnitude was significantly different during backward stepping compared to all other movement phases (initiation: *p* ≤ 0.011, forward stepping: *p* ≤ 0.013, and change of direction: *p* ≤ 0.019). Additionally, among Expert-Novice and Novice-Novice dyads, interaction force magnitude was significantly larger during initiation compared to change in direction, (*p* ≤ 0.011) (*force values are reported in previous section*).

Expert-Expert dyads used significantly larger interaction forces within movement phases of the *un*predictable PST than Expert-Novice or Novice-Novice dyads (Table 2). There were statistically significant differences in interaction force magnitude between groups during all movement phases, initiation (*F* (2,19) = 12.27, *p* < 0.0005), forward (*F* (2,19) = 43.00, *p* < 0.0005), and changes of direction (*F* (2,19) = 7.71, *p* = 0.004), backward (*F* (2,19) = 85.77, *p* < 0.0005). Tukey post-hoc testing revealed that during initiation interaction forces (mean ± SD) were significantly smaller in the EN (−6.12 ± 1.58 N, *p* = 0.005) and NN (−5.20 ± 1.41 N, *p* < 0.0005) dyads compared to EE dyads (−9.04 ± 1.53 N). There was no significant difference between EN and NN dyads, (*p* = 0.407). During forward stepping interaction forces (mean ± SD) were significant smaller in the EN (−7.38 ± 3.13 N, *p* < 0.0005) and NN (−4.06 ± 1.83 N, *p* < 0.0005) dyads compared to EE dyads (−9.04 ± 1.53 N). The EN dyads also used significantly larger interaction forces than NN dyads, (*p* = 0.021). While changing direction, interaction forces were significantly smaller in the EN (−0.87 ± 1.15 N, *p* = 0.006) and NN (−0.92 ± 0.69 N, *p* = 0.008) dyads compared to the EE dyads (−3.49 ± 1.23 N). There was no significant difference between EN and NN dyads (*p* = 0.983). Finally, during backward stepping interaction forces (mean ± SD) were significant smaller in the EN (6.83 ± 2.61 N, *p* < 0.0005) and NN (2.89 ± 1.64 N, *p* < 0.0005) dyads compared to EE dyads (14.1 ± 2.25 N). The EN dyads also used significantly larger interaction forces than NN dyads, (*p* < 0.0005).

**Table 2:** Mean interaction force per movement phase during the unpredictable partnered stepping task

	Movement phases			
	Initiation (INT)	Forward (FWD)	Backward (BKW)	Change Direction (CD)
Expert-Expert (EE)	-9.04 ± 1.53 N	-13.4 ± 2.01 N	14.1 ± 2.25 N	-3.49 ± 1.23 N
Expert-Novice (EN)	-6.12 ± 1.58N	-7.38 ± 3.13 N	6.83 ± 2.61 N	-0.87 ± 1.15 N
Novice-Novice (NN)	-5.20 ± 1.41 N	-4.06 ± 1.83 N	2.89 ± 1.64 N	-0.92 ± 1.07 N

Negative values are compression and positive values are tension

See text for significance values

Interaction forces differed between all movement phases except initiation and forward stepping among all groups during the *un*predictable PST. There were statistically significant effects of movement phase on interaction force magnitude in the EE (*F* (3,15) = 255.0, *p* < 0.0005), EN (*F* (3,21) = 114.36, *p* < 0.0005) and NN dyads (*F* (3,21) = 65.60, *p* < 0.0005). Pairwise comparisons with Bonferroni corrections revealed that regardless of group (EE, EN, and NN) interaction forces were significantly different between all movement phases (*p* ≤ 0.015) with the exception of initiation and forward stepping, *p* ≥ 0.120 (*force values reported in previous section*).

**Fig. 4:**
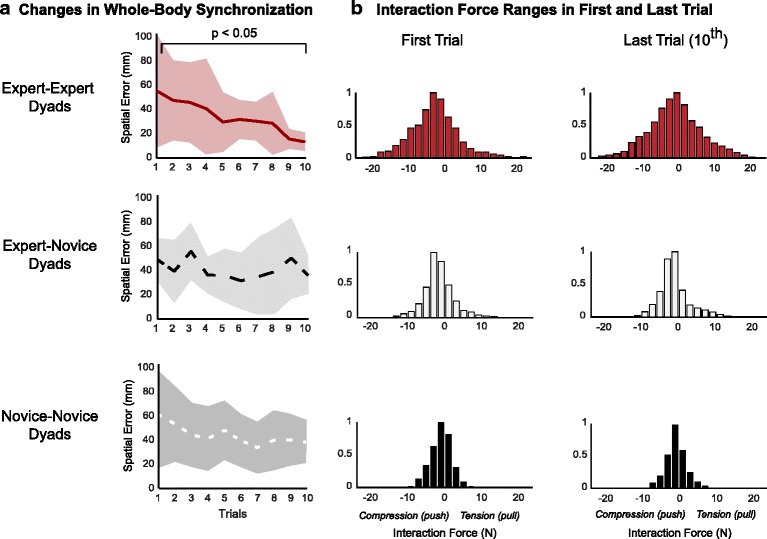
Changes in performance of the unpredictable partnered stepping task (PST) and associated interaction forces. **a** Changes in performance of the PST was quantified by the average whole-body spatial synchronization error between the leader and follower per trial. This was calculated as the mean value of the difference in the anterior-posterior position between the leader and follower with respect to the initial distance between them. The spatial synchronization error decreased from the first to last trial (i.e. performance improved) in the EE and NN dyads. However, this improvement in performance was only significant in the EE dyads (*p* < 0.05). **b** The improvements in spatial synchronization from the first to last trial of the unpredictable PST were not accompanied by changes in the range of interaction forces observed during those same trials

Improvements in performance were not associated with changes in interaction forces. The EE dyads significantly improved their performance of the PST (i.e. spatial synchronization) between the first and last trial of the unpredictable PST (mean ± SD) (first trial: 56.61 ± 41.75 mm, last trial: 13.35 ± 7.13), (*t* (5) = 2.8, *p* = 0.04). In contrast, neither the EN (first trial: 46.41 ± 13.72, last trial: 37.56 ± 14.83), (*t* (7) = 2.12, *p* = 0.07), nor NN dyads (first trial: 59.94 ± 38.59, last trial: 38.61 ± 17.19), (*t* (7) = 2.32, *p* = 0.53) demonstrated significant improvement (Fig. 4a). These improvements in performance were not accompanied by statistically significant changes in the range of interaction forces that were used during the first and last trial (mean ± SD) among EE dyads (first trial: 31.55 ± 13.68, last trial: 31.13 ± 14.48), (*t* (5) = 0.27, *p* = 0.80); EN dyads (first trial: 20.41 ± 12.19, last trial: 19.04 ± 12.62), (*t* (7) = 0.83, *p* = 0.43); or NN dyads (first trial: 12.03 ± 4.35, last trial: 10.92 ± 2.74), (*t* (7) = 0.1.61, *p* = 0.15) (Fig. 4b).

## Discussion

Our results show that physical interaction forces between two humans can be used to communicate whole-body movement goals, i.e. specify “what to do and when to do it”. Although it is known that physical guidance during walking can be provided based on interaction forces [30], this is the first characterization of the forces and how they vary according to movement goals, motor skill level, and short-term training. In contrast to prior HHI studies based on visually guided reaching, we demonstrate that proprioceptive and haptic information alone are sufficient to coordinate cooperative motor tasks, in the absence of visual and auditory information. Our work suggests that forceful interactions between human partners contain information about movement goals and their execution that can be interpreted without prior training. Additional research on other human-human interaction tasks and subjects with sensorimotor impairments is required to test the generalizability of these findings.

### Interaction force magnitude: implications and interpretations

The similarity in force magnitude across different types of physical interactions suggests that there may be a preferred physiological range of small forces used for motor communication. The interaction forces during the partnered stepping task were similar in magnitude to those measured during activities of daily living such as shaving or brushing one’s hair (5–15 N) [32]. Forces during other HHI tasks also have similar magnitude. For example, peak interaction forces between human partners range from 7 N during a visuomotor tracking task [1, 2], 15 N during a handshake [4], and 8 N when carrying an object [3]. Additionally, the interaction forces in this study were similar in magnitude to those seen during human-robot partner dancing between a human follower and robot leader [16], as well as a human leader with a robot follower [33]. In the latter of these two studies the human leaders, who were also expert partner dancers, preferred settings that allowed for interactions with the robot followers using low interaction forces. In fact, those expert leaders evaluated such settings as mimicking a better human follower than those that required higher forces [33]. While these results suggest that there may exist a preferred range for forceful interactions, the forces in these studies may be constrained by specific features of an activity such as movement speed, or the mass of an object through which partners are interacting.

Given their magnitude the interaction forces during the PST likely act primarily as force cues to communicate movement goals. The forces used by physical therapists to move the legs of a patient during body-weight supported treadmill training have been reported to be ~ 50–75 N [34]. On the other hand, interactions forces of < 1 N during interpersonal light touch provide adequate information to cause involuntary synchronization between human partners while standing and walking [35, 36]. As forces during voluntary stepping between healthy partners were on the low end between these two extremes, and as they were the only channel of communication between partners, they were likely played a greater role in communication with a smaller effect on mechanically displacing the partner. This suggests that interaction forces may be useful in motor assistance and rehabilitation without explicitly providing the energy necessary to achieve the task.

Our work directly demonstrates that that interaction forces during HHI form a communication channel, through which movement goals can be communicated and interpreted. Whereas prior studies also suggest that interaction forces create a communication channel between partners [2, 18, 19, 22], the motor activity in our experiment is different in that the only means of communication through which partners could coordinate their movements was via forces at the hands. Further, after performing a predictable stepping task, we required partners to perform a pseudorandom stepping pattern to reduce the ability of the follower to predict each step. Remarkably, all dyads –regardless of motor experience – were able to perform the unpredictable PST. Differences in the magnitude and sign of interaction forces across movement phases (Table 1) and the synchronization of movements between partners show that they were able to discern “what to do and when to do it” with sufficient accuracy based on the interaction forces alone. Further, Ganesh et al. [18] found that performance of a visuomotor tracking task was better when a bi-directional force communication channel was used versus a one-way channel where the follower could feel and respond to the actions of the leader, but the leader could not feel and respond to the actions of the follower. Bi-directional communication is likely important to obtain task-related information from the partner [18], the value of which may increase as the challenge of the cooperative motor task increases [19].

### Motor experience alters forceful interaction between human partners

The higher, more distinct interaction forces observed between expert-expert dyads could reflect more deliberate, precise, and clearer communication via forces. These higher interaction forces between experts could have arisen by stiffening the joints of the arm, a feature of partner dance that is learned in order to maintain arm posture. If the follower had stiffer arms, and resisted the mechanical effects of the forces from the leader, it would increase the forces due to a given displacement of the hand. In this way, it is possible that that experienced followers were letting the expert leader lead by amplifying force cues. Additionally, it is possible that expert followers did not move in a given direction before clearly understanding the movement goals. While a prior studies suggested that higher interaction forces between partners may indicate that the follower does not trust the leader [30], we would expect expert-expert dyads to have highest level of trust in performing the PST. Alternatively, the higher interaction forces among expert-expert dyads could arise from expert followers engaging in a greedy optimization process [37]. In this scenario, expert followers would only be concerned with the immediate cost of the next movement, causing them to “slack” [38, 39], i.e. put in less effort to predict the movement pattern, resulting in higher interaction forces for short-term energetic gain. However, contrary to our results, we would expect novices and not experts to be more likely to engage is such a strategy.

### Improvements in performance of HHI: implications for rehabilitation

The relative motor experience between partners may influence the level of performance improvement in a cooperative motor task. Improvements in performance of the PST were greatest among dyads that were matched in partner dance experience (EE and NN), but were only statistically significant among EE dyads (Fig. 4a). Similarly, improvements in performance in a seated HHI task were also greatest when partners had similar levels of experience [18]. In contrast, patients, who may be considered novices, are traditionally paired with a therapist or robot whose motor proficiency greatly exceeds that of the patient. Given the relative ineffectiveness of physical guidance under this “expert leader/novice follower” paradigm [40–42], an alternative “skill matching” approach where the skill level of the leader is matched to that of the follower may result in greater improvements in motor performance. However, under circumstance when neither patient is able to perform certain movements, such an approach may be limited. In the present study performance was evaluated as improvements over the course of training (i.e. acquisition). Given that the goal of rehabilitation is to improve independent mobility, it will be important to examine whether skill matching between partners has a similar effect when performance is assessed after a retention period, and on a motor task that can be performed individually after training with a partner. In addition to considering motor experience it may also be prudent to consider age and gender, as younger versus older, and women versus men appear to improve more during HHI [23].

The mechanisms by which performance improvements are achieved during sensorimotor cooperation with haptic interaction remain unknown [14]. We found that improvements in the PST as quantified through improved whole-body spatial synchronization were not accompanied by any changes in the magnitude of the interaction forces (Fig. 4b). This suggests that improvements in the PST could have occurred via improvements in the ability to interpret movement goals from interaction forces rather than increasing interaction force magnitude to more clearly communicate these movement goals. However, Expert-Expert dyads, the only cohort to improve with practice, used larger interaction forces. Yet these forces did not increase with practice. This suggests that Expert-Expert dyads may have started with sufficiently large interaction forces for effective communiation that the other dyads failed to reach. Therefore, improvements in HHI may involve a two-stage process; an initial increase in force to a threshold level, followed by improvements in the ability to interpret those forces with greater confidence. Additional research is required to test this hypothesis. Alternatively, improvements in the PST may have occurred as participants learned to adapt their temporal pattern of force over time, not just the spatial patterns examined here. Additional analysis beyond the scope this manuscript would be required to assess this possibility.

### Rehabilitation relevance of the partner dance paradigm

Indeed, our study was inspired by our experience with partner dance as an effective form of rehabilitation. Partner dance has been shown to improve walking and balance performance in individuals with Parkinson’s disease, stroke, and other sensorimotor and cognitive disorders [43–45]. Currently, in these rehabilitative dance scenarios, individuals with sensorimotor impairments are paired with a non-impaired human partner. Future versions of this partnered dance intervention could include a robot as the leader. However, the forces between partners during partner dance rehabilitation are not known. Therefore, we selected the PST tested here because it is a key element (i.e. a key “basic” step) of the intervention, and a reasonable task with which to begin investigating the interaction forces between human dance partners in a controlled and repeatable manner. Thus, in addition to elucidating principles that may be shared across all forms of HHI, this study sought to characterize forceful interactions specific to individuals during a PST in order to provide preliminary boundary conditions for building and designing a controller for a robot leader. Our focus on the unpredictable PST provides an upper estimate of the forces necessary for partnered walking tasks, as the ability of the follower to anticipate the characteristics of the joint movement was minimized. As shown by our data, interaction forces are lower when the follower can anticipate the intended movement goals, which may be the case in many joint movement tasks in motor assistance and rehabilitation.

### Limitations and future considerations for human-human interaction research

While our results could have important implications for human-human and human-robot interaction in rehabilitation, many key elements were not tested in this study. It should be noted that further study is required to test whether our findings are generalizable across different HHI tasks, as well as a range of cooperative, assistive, and rehabilitative movements, and different sensorimotor impairments. The novel protocol presented here could be used to examine differences in force cues necessary to guide individuals with sensorimotor impairments. Further testing is required to understand how forces vary when a member of the dyad has a sensorimotor impairment, and the degree to which the concept of “skill matching” is appropriate in such conditions. Further, the lack of auditory cues provided to the followers may have limited the ability of expert followers to take advantage of some aspects of their experience in partner dance. However, this constraint was required to ensure that only haptic cues between the hands of each partner were used to perform the PST, as opposed to additional auditory cues that would have been subject to interpretation by both the leader and the follower. The haptic cues were thus the sole means of communication of timing, i.e., when to take a step. The present study focused on the magnitude of forceful interactions. Alternative analyses such as time series analyses that assess temporal contributions of the interaction forces to communicating motor goals is required to fully identify and characterize communication channels during HHI. While we chose to focus on forces from the right hand of the leader/left hand of the follower because of their similarity upon visual inspection (Fig. 1b), it is possible that some small yet meaningful differences exist between arms during bilateral interaction tasks. Further analysis is warranted in this area. Overall, identifying principles of physical interaction based on further HHI studies could be leveraged in the design of assistive and rehabilitative robotics that are intuitive to use.

## Conclusion

Here we found that small interaction forces scaled according to motor experience may act as force cues to communicate movement goals (i.e. “what to do”). Forceful interactions between human partners may contain information about movement goals, offering a language for physical interactions. From this work, a variety of hypotheses regarding the communication of motor goals can be generated and tested. Through this testing we may be able identify principles of HHI that may guide and lead to the development of robots that can physically interact with humans in more flexible and intuitive ways, while also informing therapist-patient interactions, thereby enhancing rehabilitation outcomes.

## Abbreviations

EE: Expert-expert
EN: Expert-novice
HHI: Human-human interaction
NN: Novice-Novice
PST: Partnered stepping task

## References

[1] Reed KB, Peshkin M, Hartmann MJ (2005). Kinesthetic interaction.

[2] Reed KB, Peshkin MA (2008). Physical collaboration of human-human and human-robot teams. IEEE Trans Haptics.

[3] Ikeura R, Inooka H (1995). Cooperative force control in carrying an object by two humans.

[4] Wang Z, Yuan J, Buss M (2008). Modeling of human haptic skill. A framework and preliminary results.

[5] Marchal-Crespo L, Reinkensmeyer DJ (2009). Review of control strategies for robotic movement training after neurologic injury. J Neuroeng Rehabil.

[6] Jarrasse N, Charalambous T, Burdet E (2012). A framework to describe, analyze and generate interactive motor behaviors. PLoS One.

[7] Klingspor V, Demiris J, Kaiser M (1997). Human-robot communication and machine learning. Appl Artif Intell.

[8] Glasauer S, Huber M, Basili P, Knoll A, Brandt T (2010). Interacting in time and space: investigating human-human and human-robot joint action.

[9] Gentry S, Feron E (2005). Human-human haptic collaboration in cyclical Fitts’ tasks.

[10] Sebanz N, Bekkering H, Knoblich G (2006). Joint action: bodies and minds moving together. Trends Cogn Sci.

[11] Groten R, Hölldampf J, Peer A, Buss M (2009). Predictability of a human partner in a pursuit tracking task without haptic feedback.

[12] Reed K, Peshkin M, Hartmann MJ, Grabowecky M, Patton J, Vishton PM (2006). Haptically linked dyads: are two motor-control systems better than one?. Psychol Sci.

[13] Schubö A, Vesper C, Wiesbeck M, Stork S (2007). Movement coordination in applied human-human and human-robot interaction. HCI Usability Med.

[14] Sawers A, Ting LH (2014). Perspectives on human-human sensorimotor interactions for the design of rehabilitation robots. J Neuroeng Rehabil.

[15] Gentry S, Murray-Smith R (2003). Haptic dancing: human performance at haptic decoding with a vocabulary.

[16] Holldampf J, Peer A, Buss M (2010). Synthesis of an interactive haptic dancing partner.

[17] Rahman MM, Ikeura R (2000). Control characteristics of two humans in cooperative task and its application to robot control.

[18] Ganesh G, Takagi A, Osu R, Yoshioka T, Kawato M, Burdet E (2014). Two is better than one: physical interactions improve motor performance in humans. Sci Rep.

[19] Melendez-Calderon A, Komisar V, Burdet E (2015). Interpersonal strategies for disturbance attenuation during a rhythmic joint motor action. Physiol Behav.

[20] Wulf G, Shea CH (2002). Principles derived from the study of simple skills do not generalize to complex skill learning. Psychon Bull Rev.

[21] Cordo PJ, Gurfinkel VS (2004). Motor coordination can be fully understood only by studying complex movements. Prog Brain Res.

[22] van der Wel RPRD, Knoblich G, Sebanz N (2011). Let the force be with us: dyads exploit haptic coupling for coordination. J Exp Psychol Hum Percept Perform.

[23] Basdogan C, Ho CH, Srinivasan MA (2000). An experimental study on the role of touch in shared virtual environments. ACM Trans Comput Hum Interact.

[24] Feth D, Groten R, Peer A, Hirche S (2009). Performance related energy exchange in haptic human-human interaction in a shared virtual object manipulation task.

[25] Sallnäs EL, Zhai S (2003). Collaboration meets Fitts’ Law: passing virtual objects with and without haptic force feedback.

[26] Groten R, Feth D, Peer A, Buss M (2009). Efficiency analysis in a collaborative task with reciprocal haptic feedback.

[27] Reed KB, Peshkin M, Hartmann MJ (2006). Haptic cooperation between people, and between people and machines.

[28] Stefanov N, Peer A, Buss M (2009). Role determination in human-Human interaction.

[29] Ikeura R, Morita A, Mizutani K (1997). Variable damping characteristics in carrying an object by two humans.

[30] Ranasinghe A, Dasgupta P, Althoefer K, Nanayakkara T (2015). Identification of haptic based guiding using hard reins. PLoS One.

[31] Krampe RT, Ericsson KA (1996). Maintaining excellence: deliberate practice and elite performance in young and older pianists. J Exp Psychol Gen.

[32] Hawkins KP, King C-H, Chen TL, Kemp CC (2012). Informing assistive robots with models of contact forces from able-bodied face wiping and shaving.

[33] Chen TL, Bhattacharjee T, McKay JL, Borinski JE, Hackney ME, Ting LH (2015). Evaluation by expert dancers of a robot that performs partnered stepping via haptic interaction. PLoS ONE.

[34] Galvez JA, Budovitch A, Harkema SJ, Reinkensmeyer DJ (2011). Trainer variability during step training after spinal cord injury: Implications for robotic gait-training device design. JRRD.

[35] Johannsen L, Guzman-Garcia A, Wing AM (2009). Interpersonal light touch assists balance in the elderly. J Mot Behav.

[36] Zivotofsky AZ, Hausdorff JM (2007). The sensory feedback mechanisms enabling couples to walk synchronously: An initial investigation. J Neuroeng Rehabil.

[37] Emken JL, Benitez R, Reinkensmeyer DJ (2007). Human-robot cooperative movement training: learning a novel sensory motor transformation during walking with robotic assistance-as-needed. J Neuroeng Rehabil.

[38] Reinkensmeyer DJ, Patton JL (2009). Can robots help the learning of skilled actions?. Exerc Sport Sci Rev.

[39] Reinkensmeyer DJ, Emken JL, Cramer SC (2004). Robotics, motor learning, and neurologic recovery. Annu Rev Biomed Eng.

[40] Armstrong TR (1970). Training for the production of memorized movement patterns, PhD thesis.

[41] Sidaway B, Ahn S, Boldeau P, Griffin S, Noyes B, Pelletier K (2008). A comparison of manual guidance and knowledge of results in the learning of a weight-bearing skill. J Neurol Phys Ther.

[42] Winstein CJ, Pohl PS, Lewthwaite R (1994). Effects of physical guidance and knowledge of results on motor learning: support for the guidance hypothesis. Res Q Exerc Sport.

[43] Hackney ME, Hall CD, Echt KV, Wolf SL (2012). Application of adapted tango as therapeutic intervention for patients with chronic stroke. J Geriatr Phys Ther.

[44] Hackney ME, Earhart GM (2010). Effects of dance on gait and balance in Parkinson’s disease: a comparison of partnered and nonpartnered dance movement. Neurorehabil Neural Repair.

[45] Hackney ME, Earhart GM (2010). Social partnered dance for people with serious and persistent mental illness. J Nerv Ment Dis.

